# The RepVig framework for designing use-case specific representative vignettes and evaluating triage accuracy of laypeople and symptom assessment applications

**DOI:** 10.1038/s41598-024-83844-z

**Published:** 2024-12-23

**Authors:** Marvin Kopka, Hendrik Napierala, Martin Privoznik, Desislava Sapunova, Sizhuo Zhang, Markus A. Feufel

**Affiliations:** 1 Division of Ergonomics, Department of Psychology and Ergonomics (IPA), Technische Universität Berlin, Berlin, Germany; 2 Institute of General Practice and Family Medicine, Charité – Universitätsmedizin, Corporate Member of Freie Universität Berlin and Humboldt- Universität zu Berlin, Berlin, Germany; 3 Emergency and Acute Medicine and Health Services Research in Emergency Medicine, Charité – Universitätsmedizin, Corporate Member of Freie Universität Berlin and Humboldt- Universität zu Berlin, Berlin, Germany

**Keywords:** Health services, Public health

## Abstract

Most studies evaluating symptom-assessment applications (SAAs) rely on a common set of case vignettes that are authored by clinicians and devoid of context, which may be representative of clinical settings but not of situations where patients use SAAs. Assuming the use case of self-triage, we used representative design principles to sample case vignettes from online platforms where patients describe their symptoms to obtain professional advice and compared triage performance of laypeople, SAAs (e.g., WebMD or NHS 111), and Large Language Models (LLMs, e.g., GPT-4 or Claude) on representative versus standard vignettes. We found performance differences in all three groups depending on vignette type: When using representative vignettes, accuracy was higher (OR = 1.52 to 2.00, *p* < .001 to .03 in binary decisions, i.e., correct or incorrect), safety was higher (OR = 1.81 to 3.41, *p* < .001 to .002 in binary decisions, i.e., safe or unsafe), and the inclination to overtriage was also higher (OR = 1.80 to 2.66, *p* < .001 to *p* = .035 in binary decisions, overtriage or undertriage error). Additionally, we found changed rankings of best-performing SAAs and LLMs. Based on these results, we argue that our representative vignette sampling approach (that we call the RepVig Framework) should replace the practice of using a fixed vignette set as standard for SAA evaluation studies.

## Introduction

Symptom-assessment applications (SAAs) are digital health tools that assist medical laypeople in self-diagnosing and determining whether and where to seek health care (self-triage)^1,2^. Whereas some research in this domain focuses on how SAAs impact health systems or on how to improve their usability and user experience^2–7^, a significant portion of studies investigates the accuracy of SAAs^8,9^. This line of research started with Semigran et al. in 2015,^10^ who developed 45 case vignettes to systematically test and compare the accuracy of SAAs, see Table 1. These vignettes were developed by clinicians, drawing from a variety of medical resources including textbooks for medical education. Subsequently, numerous studies have adopted their methodology and/or vignettes. Some authors have used the same set of vignettes^11–13^, whereas others have developed their own set, either building on the original vignettes or employing a similar approach to create them^14–16^. Over time, these case vignettes have faced various criticisms: in addition to procedural criticism (e.g., that it matters who inputs vignettes into SAAs and that interrater reliabilities should be calculated^17^), most criticism refers to the content of the case vignettes (e.g., the symptoms and symptom clusters covered in the cases)^8^ or to how they were created (e.g., the vignettes are usually created by clinicians, who may describe symptoms differently than patients and have access to physical examination results, and the vignettes are often fictitious rather than based on real cases)^18^.

**Table 1:** Examples of traditionally used case vignettes by Semigran et al.^10^.

Author	Example Case Vignette 1	Example Case Vignette 2
Semigran et al	A 67-year-old woman with a history of COPD presents with 3 days of worsening dyspnea and increased frequency of coughing. Her cough is now productive of green, purulent sputum. The patient has a 100-pack-year history of smoking. She has had intermittent, low-grade fever of 100°F (37.7 °C) for the past 3 days and her appetite is poor. She has required increased use of rescue bronchodilator therapy in addition to her maintenance medications to control symptoms	A 30-year-old man presents with a painful, swollen right eye for the past day. He reports minor pain on palpation of the eyelid and denies any history of trauma, crusting, or change in vision. He has no history of allergies or any eye conditions and denies the use of any new soaps, lotions, or creams. On exam, he has localized tenderness to palpation and erythema on the midline of the lower eyelid near the lid margin. The remainder of the physical exam, including the globe, is normal

To mitigate these issues, SAAs were tested with cases that more closely resemble real-world situations and actual patients. For instance, Yu et al.^19^ and Berry et al.^20^ utilized patient data from Emergency Department (ED) presentations for their vignettes. This method enhances external validity – understood as the applicability of findings to real-world scenarios^21^ – yet its representativeness is still limited with respect to the primary purpose of SAAs: aiding medical laypeople experiencing acute symptoms deciding if or where to seek care^22^. That is, these vignettes include problems of people who chose to visit an ED, a group that may not fully represent the wider array of SAA users who (rightly or wrongly) decided against going to the ED in the first place. Additionally, the case development was biased, as clinicians re-created these vignettes based on their documentation, who may have inadvertently filtered information or phrased the vignettes differently than laypeople using an SAA would have done^18^. Assuming the use case of self-triage (deciding if and where to seek care), previous studies thus did not yet generate a representative set of vignettes. Results of SAA audit studies that are based on such stimuli might not be generalizable to laypeople’s self-triage with SAA in the real world in which laypeople typically enter the symptoms. Because SAAs are often evaluated for validation purposes to demonstrate that they can handle a percentage of cases they are approached with in the real world, it is crucial to use vignettes that are representative of these situations. Although using theoretically derived symptom patterns (e.g., atypical presentations) might be another use case for SAA evaluations, they have not been the focus of existing SAA research yet^8,9^.

**Fig. 1:**
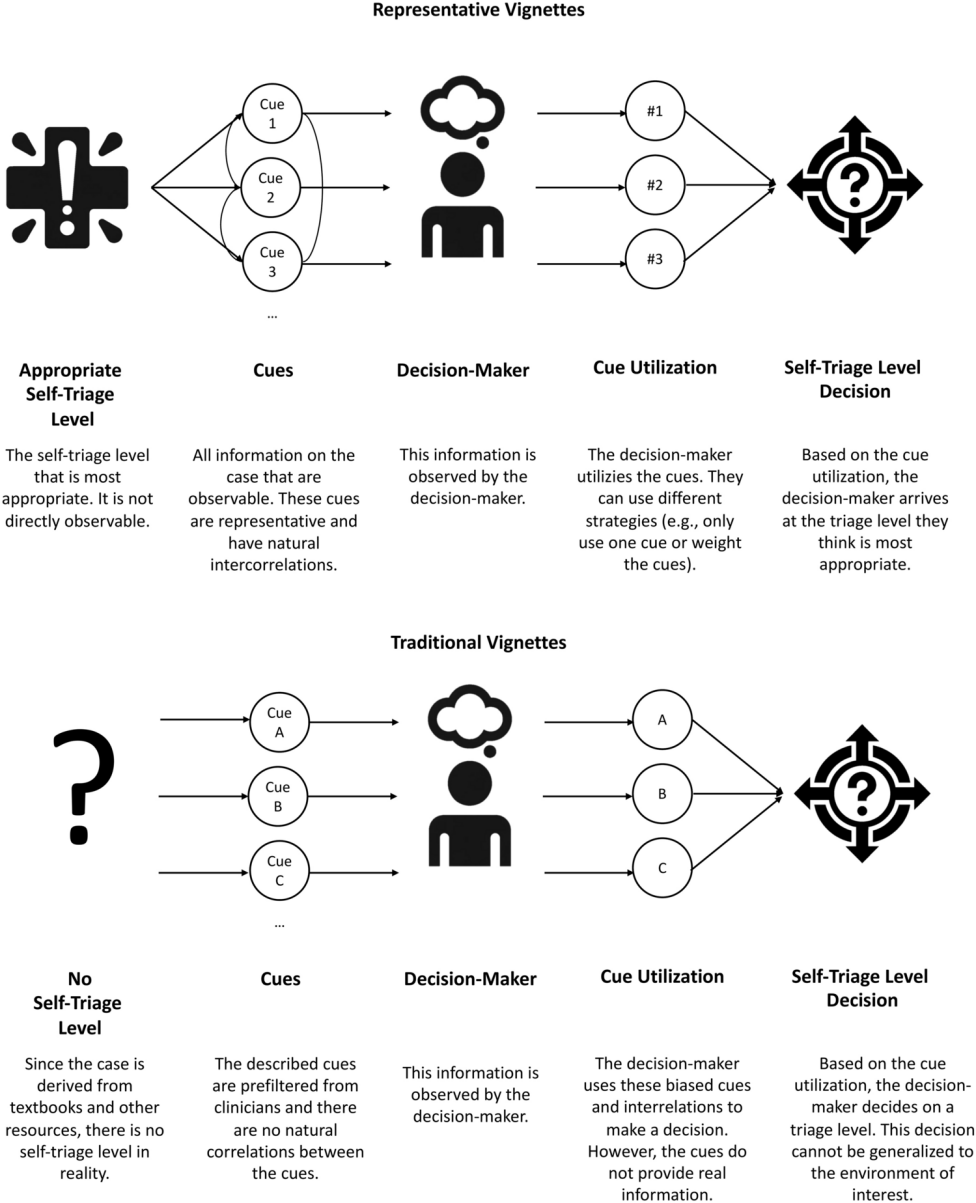
Self-triage lens model.

The conceptual challenge of generating a representative set of stimuli was recognized in decision-making research decades ago, particularly in the field of ecological psychology rooted in Egon Brunswik’s probabilistic functionalism^23^. It proposes that relationships between environmental information and objects are probabilistic in nature. In his framework, decision-makers infer unobservable conditions (“latent variables” or a triage level) from a set of observable information (“cues” or symptoms) based on characteristic correlations between certain cues and the latent variable (see a previously published article on Brunswik’s probabilistic functionalism^21^ for an overview). Figure 1 implements these considerations for self-triage decisions.

Based on these considerations, Brunswik coined the term *ecological validity* and proposed the concept of *representative design* as a method for sampling ecologically valid stimuli. Using representative design, stimuli (or vignettes) are sampled directly from situations to which the researcher would like to generalize, thereby trying to maintain the natural correlations between cues and the latent variable^23^. This approach contrasts starkly with the traditional *systematic design*, where parameters of the stimuli are systematically varied, which according to representative design may lead to biased cues and intercorrelations^21^. For example, a higher age might be associated with a higher risk for certain diseases, and systematically varying age tends to eliminate these associations. As a result, decision-makers might make different decisions when given systematically designed stimuli, because the correlations between the described cues are not natural or representative anymore. Thus, previous research on participants’ self-triage decision making could be biased because participants might have made decisions differently in lab settings that in the real world. For example, Gigerenzer et al. compared ecologically valid and systematically varied stimuli and found that decision-making strategies differed between these stimuli, because humans often use only few cues in their decision making^24^. Applied to a self-triage use case, a person experiencing severe pain (10 out of 10) would not seek additional information and visit the emergency department based on this information alone. If the pain is – according to systematic design principles – changed to 2 out of 10, participants might use other decision-making strategies and not rely solely on pain as the most important information but also include additional cues. Another example of the influence of interrelations between cues is myocardial infarctions. Given the varying prevalences of myocardial infarctions by age^25^, shortness of breath and chest pain are most likely signs of a myocardial infarction among 85-year old patients, whereas the same symptoms among 20-year-old patients most likely indicate a panic attack. The correct self-triage level and also the decisions participants make for these two cases might thus be different. These examples demonstrate that systematic design can lead to artificial vignettes and decisions that differ from those made in the real world. Thus, stimuli based on representative design help researchers induce and evaluate decision-making behavior that more readily generalizes to the environment of interest, in our case, from SAA audit studies to real-world self-triage performance of medical laypeople. However, it should be noted that while representative design enhances ecological validity, it may have limited explainability compared to systematic design, because natural correlations between cues and the latent variable are retained and thus effects might be more challenging to explain and interpret.

The power of representative design has been demonstrated in various fields such as pharmacy^26^, social psychology^27^, cognitive science^28^, human resources^29^, and public health^30^, showing that predictive power of results from experiments increases and these results better resemble real-world performance. Studies that compared representative stimuli with non-representative stimuli found significant differences between results and conclusions^24,27,29,31^. For example, ecologically valid stimuli tended to make phenomena identified with non-representative stimuli disappear or reverse the direction of effects^27^ and better predicted performance in field studies^29,31^.

To our knowledge, however, the representative design framework has thus far not been applied to study self-triage decisions and to evaluate SAA performance. Our paper aims to bridge this research gap. Building on an application of the representative design approach to the context of self-triage decisions, we explore how a framework to generate representative vignettes (that we called the RepVig Framework) can be used to effectively generate new vignettes to assess self-triage capabilities. Our study assesses the following research question: How do performance estimates of laypeople, SAAs, and LLMs derived from representative vignettes differ from those derived from traditional vignettes?

Both vignette types have their strengths and weaknesses. Traditional vignettes are often constructed post hoc from expert knowledge or clear medical cases and might be useful when performance needs to be estimated with minimal resources. They are also beneficial for testing specific hypotheses about SAAs when information should be varied, e.g., to examine whether adding an additional symptom (to make clinical presentations atypical) yields different results. However, their generalizability is limited, and they may not accurately predict the average performance in the real world. In contrast, representative vignettes aim to capture the real-world complexity and variability of symptoms as experienced by SAA users. Given that SAAs are developed for medical laypeople deciding whether and where to seek care, representative vignettes are better suited for generalizing results to internet or app users making these decisions and seeking assistance in their decision-making.

Whereas traditional vignettes are valuable for evaluating SAA performance for a theoretically defined range of symptoms, representative vignettes are more appropriate for assessing how these tools might perform in real-world settings where correct solutions are often unknown. Since previous studies tried to validate specific tools and aimed to estimate how well apps perform – on average – in the real world^8–10^, it is important to use representative vignettes that can predict real-world performance. Although this is the most commonly researched use case, researchers should always specify the goal of their study and the use case they examine these tools for.

Based on these considerations, we compare results obtained from the representative vignettes (developed for the actual SAA real-world use case) that we developed using the RepVig Framework with those developed with traditional approaches and test whether these results differ. This comparison allows testing whether previous studies may have misjudged the average performance of laypeople, SAAs, and LLMs in the real world due to non-representative vignettes. We hypothesize that these two methods of vignette development will yield different results.

## Methods

### Ethical considerations

This study was carried out in accordance with the declaration of Helsinki and all participants provided informed consent to participate in the study. Ethical approval was granted by the ethics committee of the Department of Psychology and Ergonomics (IPA) at Technische Universität Berlin (tracking number: AWB_KOP_2_230711). The study was preregistered in the WHO-accredited German Clinical Trials Register (ID: DRKS00032895). The methods and results are reported conforming to the STROBE reporting guideline^32^ and metrics are calculated as reported in the symptomcheckR study^33^.

### Study design

This study was designed as a prospective observational study examining the (self-)triage performance of medical laypeople, SAAs and Large Language Models (LLMs) based on two kinds of vignettes. We used a set of existing vignettes that have been used to evaluate triage performance of laypeople and SAAs in previous studies^34,35^. In addition, we aimed to develop a new vignette set following a representative design approach^23^ and the RepVig Framework, aiming for greater external validity in generalizing to self-triage decisions compared to prior studies. We investigated whether vignettes obtained using the RepVig framework yield different triage performance estimates compared to the traditional vignette sets. We gathered urgency assessments from medical laypeople and entered the vignettes in various SAAs and LLMs to determine standard metrics for triage-performance reporting^33,36^.

### Vignette sets

Brunswik^23^ emphasized the importance of sampling stimuli (vignettes in this case) from a population of stimuli from the reference class. To gather such symptom descriptions from individuals who are (1) self-describing their symptoms and (2) in the process of deciding if and where to seek healthcare, we utilized the social media network Reddit, specifically the subreddit 'r/AskDocs’. This forum allows people to post medical questions, which are then addressed by verified physicians on a voluntary basis. We considered these descriptions approximations of real-world scenarios in which laypeople made self-triage decisions, because online users being in the situation of deciding whether and where to seek care posted them. Posts on this forum had the benefit of including symptoms from a layperson’s perspective without including additional information that laypeople do not have access to when making these decisions (e.g., results from physical examinations or lab tests). The posts also ensured that cases are phrased the way laypeople would describe them. For our research, we used Reddit’s API to extract all new posts within a 14-day period, from June 16th to June 29th 2023.

Recognizing that the symptom distribution in these cases might differ from those typically entered into SAAs, we applied Brunswik’s second approach to achieving representative stimuli, known as ‘canvassing’^21^. To this end, we established quotas based on symptom clusters commonly input into SAAs. We used data from a previous study^37^ that examined symptom clusters based on the CDC’s National Ambulatory Medical Care Survey (and corresponding triage advice) that users entered into an SAA, and constructed these quotas to ensure that our vignette set is representative not only in terms of real-world symptom descriptions but also with respect to the symptom distributions that users tend to enter into SAAs. These quotas can be found in Table 2.

**Table 2:** Quotas used to sample vignettes that are representative of symptom clusters typically entered in SAA based on a previous study on symptom distributions^37^.

Symptom Cluster	# of vignettes (%)
Musculoskeletal Pain	5 (11%)
Joint Pain	3 (7%)
Headache	1 (2%)
Chest Pain	1 (2%)
Other Pain	5 (11%)
Gynecological	5 (11%)
Tumors/lumps/masses	4 (9%)
Edema	2 (4%)
Skin issues	2 (4%)
Gastrointestinal	2 (4%)
Impaired sensations	3 (7%)
Urinary Tract Problems	3 (7%)
Upper Respiratory Symptoms	1 (2%)
Other	8 (18%)
Total Number	45 (100%)

To ensure that we include vignettes by people who are experiencing a situation in which they would consult an SAA (i.e., that they experienced new symptoms and sought advice on what to do), we applied the following exclusion criteria, omitting vignettes where authors:

- Sought general information only,
- Provided a picture to be understandable, as these cannot be entered into SAAs,
- Provided overly specific information (e.g., blood values that are typically not known to laypeople),
- Discussed symptoms for which the individual had already consulted a medical professional,
- Provided insufficient details (e.g., a single sentence stating foot pain) for self-triage,
- Described symptoms experienced by someone else (with the exception of infants),
- Did not describe acute symptoms (e.g., past symptoms that have already resolved)
- Described symptoms already diagnosed by a medical professional
- Exceeded 1,000 characters (which may overwhelm participants).

During the 14-day sampling period, this left us with 8,794 vignettes. Using a selection method based on R and Shiny Dashboard, we randomly drew one vignette at a time and manually either included it in the relevant quota or excluded it based on our predefined exclusion criteria. When a specific quota was filled, any new vignettes in that category were excluded. We continued this procedure until all quotas were filled. Once we compiled our vignette set, we made minor adjustments, such as correcting typographical errors and standardizing units of measurement (e.g., adding “kg” to vignettes that originally reported weights in “lbs”). We did not edit the vignettes in any other way, because the principles of representative design require stimuli to be as close to the actual decision environment as possible. Any modification could unpredictably affect the cues and their correlations to self-triage decisions and thus the representativeness of the vignettes. Using this sampling method, the RepVig Framework, we compiled a final set of 45 vignettes to construct a set that is comparable in size to previous studies^10,14^.

In alignment with established best practices and previous SAA research^14,16,38^, two licensed physicians assessed the appropriate triage level (i.e., if the symptoms require emergency care, non-emergency care, or self-care) of each vignette independently. After rating all vignettes, the physicians discussed their answers in cases of disagreement to reach consensus on the appropriate triage level, thereby finalizing the solutions for this set. This approach guarantees that the triage levels assigned to each vignette are not only based on expert medical opinion but also on a harmonized understanding between the two assessing physicians^14,16,38^.

For the evaluation study, we compared data collected with our novel sampling approach to data collected with vignettes developed by Semigran et al.^10^. This dataset was selected for comparison, because it is openly accessible and has been used to evaluate the capabilities of both medical laypeople and SAAs. The dataset focusing on laypeople’s capability^34^ was published in 2021 and comprises data from 91 participants, each of whom rated all 45 vignettes developed by Semigran et al. The dataset assessing SAA accuracy^35^ replicated an earlier accuracy study by utilizing the same set of vignettes and SAA selection criteria. The authors tested 22 different SAAs using the same set of 45 vignettes from Semigran et al. that were used in the study examining laypeople’s capabilities.

### Data collection

To compare triage performance with both vignette sets, we collected data from three different groups: Medical laypeople, SAAs and LLMs. To obtain data from laypeople, we used Prolific, an online panel provider known for its high-quality data^39^. We drew a random sample from this platform, including participants from the United States (to ensure comparability with our comparator dataset) and excluding participants that were health professionals. Based on our comparator dataset^40^, we aimed to get about 4,000 ratings to arrive at a similar number of ratings as in the comparator dataset (4,095 ratings) to ensure comparability and adequate statistical power to detect effects of similar size under similar conditions. To ensure attentive participation, each person was assigned a random sample of 20 out of the 45 vignettes. Thus, we collected data from 202 participants between the October 18^th^ and 19^th^ 2023 using an online survey designed with Unipark (Questback)^41^. Based on Levine et al.’s easy-to-comprehend and laypeople-friendly phrasing of different urgency levels^12^, participants were asked to choose whether the most appropriate level for each vignette is emergency care, urgent care, non-emergent care or self-care. To further motivate patients, we offered a bonus of 0.02$ for each correctly solved vignette, in addition to a base remuneration of 1$ for 10 min. Additionally, we embedded an attention check in two fictitious vignettes, instructing participants to select a specific response. Data from participants who failed to pass at least one attention check were excluded from the analysis.

Following best practices to reduce effects from inputter variability, data from SAAs were collected by two graduate students (DS and SZ) – without professional medical training – who entered the cases into different SAAs between October 24^th^ 2023 and January 22^nd^ 2024. Having students enter the vignettes rather than health professionals ensured that only information stated in the vignettes were entered without subjective clinical interpretation of symptoms, which could introduce bias^17^. The two students entered each vignette into 12 different SAAs (amounting to 540 assessments). The selection of SAAs was based on our comparator dataset^13^ to ensure comparability. The original study from which this dataset is derived had systematically identified all publicly available SAAs. For our study, we had to exclude certain SAAs because four were no longer available, two discontinued providing triage advice, two had shifted to a subscription model, and two now give specialized advice for pediatric cases. A comprehensive list of the remaining 12 SAAs is provided in the Appendix. Inputters were blinded to the outcome (i.e. the correct solution) and recoded the triage levels provided by the SAAs. These results were then reviewed by the lead author (MK). In instances where the inputters came up with different results, we considered a vignette correctly solved if at least one of them had recorded the correct solution. This criterion was based on the rationale that if one inputter could derive the correct solution, the SAA was technically capable of providing an accurate triage assessment^42^. Analyses for each inputter separately can be found in the Appendix.

Data from LLMs were collected by one person only between November 28^th^ and 29^th^ 2023, as we used a predefined, one-shot prompt for all LLMs to circumvent input variation. We collected data for both the vignettes from our comparator dataset and those developed in this study. The prompt we used was adapted from two previous studies^12,43^ and stated.

*“Please give a triage classification for the following vignette. The criteria for triage classifications are presented below: Emergency: Call 911 or go directly to the emergency room; Non-Emergency: Try to see a doctor, but the symptoms do not require immediate attention at an emergency room. For example, you can see a doctor in the next week. Self-care: Let the health issue get better on its own and review the situation in a few days again.”.*

We tested all LLMs that we could identify through a systematic search (entering combinations of the words “large language model”, “LLM”, “chatbot”, “GPT”, “text generation” and “BERT” into search engines) and that offered a web interface. In total, we evaluated five LLMs. The list of LLMs can be found in the Appendix.

### Data analysis

To align our study with the comparator datasets, which did not include ‘urgent care’ as a distinct solution, we recoded all ‘urgent care’ responses to 'non-emergency care’. This adjustment ensures consistency in our analysis, as ‘urgent care’ was originally classified as 'non-emergency care’ in the comparator datasets.

We then proceeded to compare the entire set of vignettes obtained through our novel sampling approach with the vignette set commonly used in previous studies assessing SAA accuracy^10^. For the analysis involving medical laypeople, SAAs and LLMs, we employed both descriptive and inferential statistical methods. For our descriptive analysis, we reported mean (M) and standard deviation (SD) of all metrics to describe the data and the variability around the mean to demonstrate the spread of the performance metrics. To examine the differences between our newly sampled vignette set and the traditional set used for laypeople and SAAs, we used mixed-effects binomial logistic regressions in our inferential analysis. In these models, participants (or SAAs or LLMs) were treated as a random effect, while the vignette sets were considered a fixed effect. The model was specified as follows:

$$P\left({Y}_{ij}=1\right)= \frac{1}{1+{e}^{-({\beta }_{0}+{C}_{i}{\beta }_{1}+ {\alpha }_{j})}}$$

where *P(Y*_*i*_ = *1)* represents the probability of solving the vignette correctly, *β*_*0*_ and *β*_*1*_ denote the coefficients, *C*_*i*_ whether the case is from the representative or traditional vignette set, and *α*_*j*_ the random effect of the participant (or SAA or LLM) assessing the vignette.

Our study’s outcome measures were aligned with established reporting guidelines that synthesized commonly reported metrics in SAA research and standardized them (symptomcheckR)^36^. These metrics included overall accuracy, accuracy for each self-triage level separately, the safety of advice (calculated as the percentage of emergencies identified), the comprehensiveness (how many of the total number of vignettes received a solution), and the inclination to overtriage (the percentage of overtriage errors among all errors). Since not all SAAs gave advice for each vignette, we calculated a Capability Comparison Score (CCS, as elaborated in a previous study on this metric^36^). This metric adjusts the accuracy scores to reflect the varying difficulty levels of vignettes that different SAAs were able to address. Cases that are more challenging to solve have a higher impact on this score, while easier cases have a lower impact. Although this metric does not account for the different triage levels, it considers the number of cases each SAA solved and makes the performance comparable by controlling for item difficulty and cases that an SAA did not provide advice to. This allows for a more equitable comparison of capability across SAAs that may not have provided solutions for all cases. For assessing the interrater agreement on SAA data, we used two-way mixed, agreement, average-measures intra-class correlation (ICC), as solutions were coded as ordinal^44^. Values above 0.40 were considered acceptable^45^.

## Results

### Vignette sampling

**Fig. 2:**
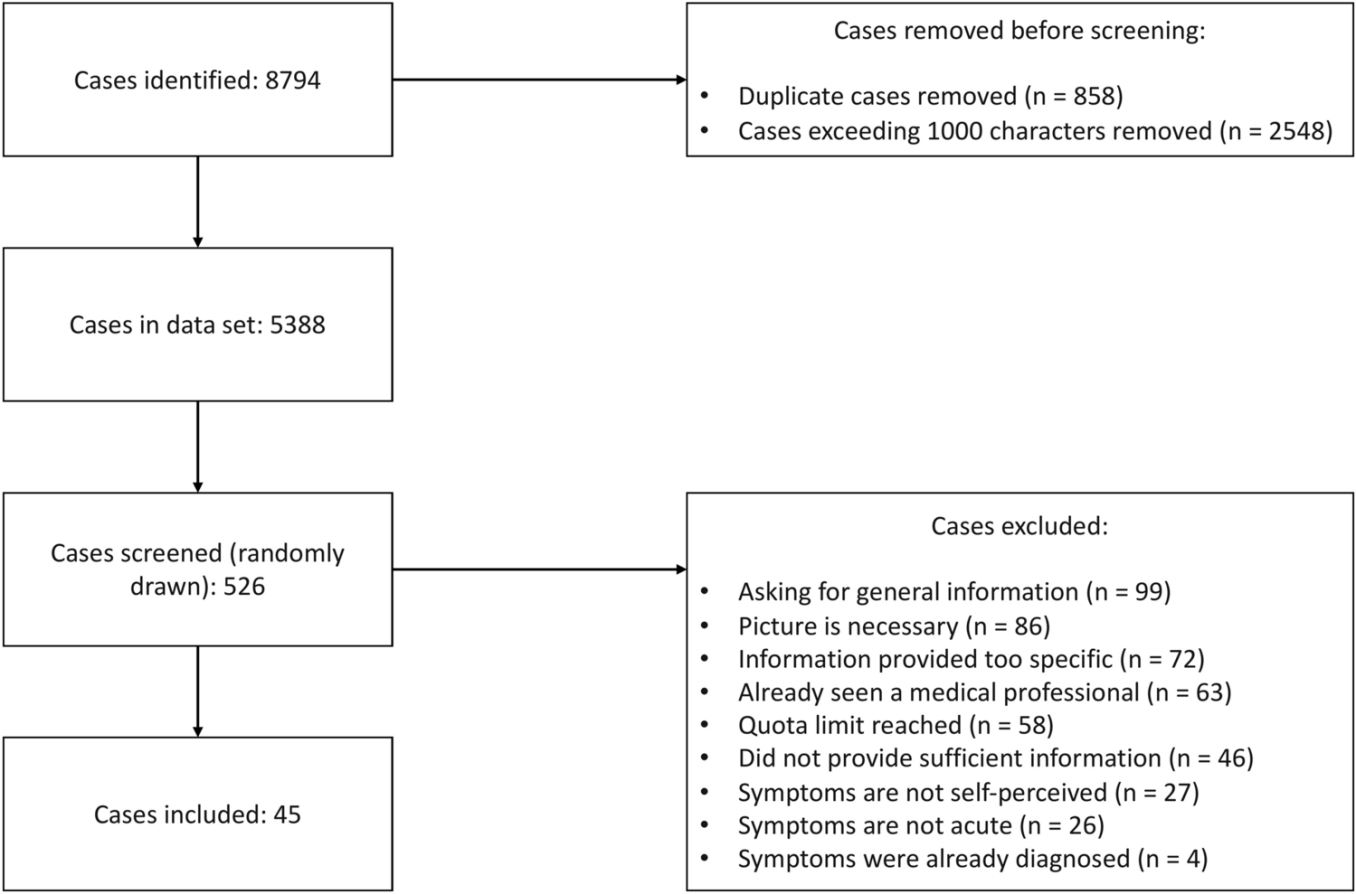
PRISMA chart with number of case vignettes identified, screened, excluded and included.

The vignette sampling process is displayed in Fig. 2 based on the PRISMA reporting standard^46^. Over a 14-day period, we identified 8,794 posts, of which 858 were duplicates. After removing cases that were too long, we arrived at a final set of 5,388 posts. We randomly sampled from these posts until all quotas were filled, reviewing 526 vignettes in total. Out of these, 423 were excluded based on the exclusion criteria and 58 because quota limits were reached.

### Participants

Out of the 33,736 eligible participants on the Prolific platform, 204 participants started the survey and 202 completed it. Data from four participants were excluded, because they failed at least one attention check. This resulted in a final sample size of 198 participants with 20 assessments each and a total number of 3,960 vignette assessments. Descriptions of participants’ characteristics can be found in Table 3.

**Table 3:** Description of participants. N = 198, M = Mean, SD = Standard Deviation, n = number.

Characteristic	Result
**Age**, M (SD)	40.70 (13.85)
**Gender**, n (%)	
Male	97 (48.99%)
Female	98 (49.49%)
Other	3 (1.52%)
**Education**, n (%)	
Less than high school diploma	2 (1.01%)
High school graduate, GED, or alternative	29 (14.46%)
Some college or Associate degree	61 (30.81%)
Bachelor degree	73 (36.87%)
Graduate degree or higher	33 (16.67%)
**Medical training**, n (%)	
No training at all	165 (83.33%)
Basic first aid training	33 (16.67%)
**eHealth Literacy** (quantified by the eHealth Literacy Scale), M (SD)	30.4 (4.37)

### Self-triage performance metrics

#### Laypeople’s performance

On average, there was no performance difference for laypeople triaging vignettes sampled for this study and vignettes that were traditionally used (OR = 0.94, SE = 0.05, z = -1.40, p. = 0.16). However, laypeople identified emergency cases more often using representative vignettes compared to traditional vignettes (OR = 1.77, SE = 0.21, z = 2.72, *p* = 0.006). The same pattern emerged for non-emergency cases (OR = 1.27, SE = 0.08, z = 2.94, *p* = 0.003). Conversely, laypeople identified self-care cases less often with representative than with traditional vignettes (OR = 0.59, SE = 0.10, z = -5.08, *p* < 0.001). As a result, the estimated safety of advice based on representative vignettes can be considered higher than based on traditional vignettes (OR = 1.84, SE = 0.08, z = 7.38, *p* < 0.001). Overall, laypeople were more inclined to overtriage with representative vignettes compared to traditional vignettes (OR = 2.10, SE = 0.10, z = 7.43, *p* < 0.001). A summary of these findings can be found in Table 4.

**Table 4:** Laypeople’s self-triage performance with representative versus traditional vignettes, M = mean, SD = standard deviation.

Metric	Representative Vignettes, M(SD)	Traditional Vignettes, M(SD)	OR	p Value
Average Accuracy	62.4 (10.80)	60.9 (6.81)	0.94	.16
Emergency Accuracy	78.6 (37.6)	67.5 (16.4)	1.77	.006
Non-Emergency Accuracy	73.2 (13.0)	68.4 (13.8)	1.27	.003
Self-Care Accuracy	34.6 (24.4)	46.7 (15.9)	0.59	< .001
Safety of Advice	90.7 (7.9)	84.2 (8.2)	1.84	< .001
Inclination to overtriage	74.6 (20.4)	60.1 (17.6)	2.10	< .001

#### SAA performance

Agreement between both SAA inputters was acceptable (ICC = 0.605). Across all SAAs, triage accuracy was significantly higher for representative vignettes compared to traditional vignettes (OR = 2.00, SE = 0.139, z = 5.68, *p* < 0.001). In detecting emergencies, we could not find a statistically significant difference (OR = 2.26, SE = 0.53, z = 1.52, *p* = 0.13), but SAAs detected non-emergency cases (OR = 2.38, SE = 0.236, z = 3.70, *p* < 0.001) and self-care cases more often (OR = 2.53, SE = 0.292, z = 3.18, *p* = 0.0015) in representative vignettes compared to traditional vignettes. The safety of advice was higher with representative vignettes for one inputter (OR = 1.81, SE = 0.224, z = 2.64, *p* = 0.008), and we found a similar but non-significant trend for the second inputter (OR = 1.42, SE = 0.206, z = 1.70, *p* = 0.09). With representative vignettes, the inclination to overtriage was higher for one inputter (OR = 1.80, SE = 0.277, z = 2.112, *p* = 0.035) and lower for the other inputter (OR = 0.53, SE = 0.277, z = -2.30, *p* = 0.022). See Table 5 for a summary.

**Table 5:** SAA’s self-triage performance with representative versus traditional vignettes, M = mean, SD = standard deviation.

Metric	Representative Vignettes, M(SD)	Traditional Vignettes, M(SD)	OR	p Value
Average Accuracy	67.8 (46.8)	48.9 (50.0)	2.00	< .001
Emergency Accuracy	75.0 (44.2)	54.4 (49.9)	2.26	.013
Non-Emergency Accuracy	76.4 (42.5)	60.6 (49.0)	2.38	< .001
Self-Care Accuracy	46.8 (50.1)	31.7 (46.6)	2.53	.002
Safety of Advice*	83.7 (11.1) 92.2 (5.3)	85.6 (8.1)	1.81 / 1.42	.008 / .09
Inclination to overtriage*	52.5 (29.0) 74.0 (17.6)	56.6 (23.8)	1.80 / 0.53	.035 / .022

* Since no aggregated statistic can be calculated for this metric, values for both inputters are reported.

The average item difficulty for representative vignettes among SAAs was higher (M = 0.68, SD = 0.21) than for the traditional vignettes (M = 0.49, SD = 0.26). Performance comparisons with CCS values and ranks are summarized in Table 6. Detailed metrics for every individual SAA can be found in the Appendix.

**Table 6:** Capability comparison scores (CCS) and ranks for SAAs in both vignette sets.

Symptom-Assessment Application	Capability Comparison Score in representative vignette set	Capability Comparison Score in traditional vignette set	Rank in representative vignette set	Rank in traditional vignette set
Healthwise	56.1	52.2	1	5
NHS 111	56.1	47.8	1	8
Everyday Health	55.0	42.2	3	11
Symptomate	55.0	42.2	3	11
Healthdirect	53.9	46.7	5	9
Ada	52.8	57.8	6	2
Isabel	50.6	53.3	7	3
Drugs.com	50.6	58.9	7	1
NHS	47.2	45.6	9	10
Family Doctor	41.7	41.1	10	6
Symptify	41.7	53.3	10	3
Healthily	39.4	48.9	12	7

#### LLM performance

Across all LLMs, triage accuracy was significantly higher for representative vignettes compared to traditional vignettes (OR = 1.52, SE = 0.196, z = 2.14, *p* = 0.03). In detecting emergencies, we could not find a statistically significant difference (OR = 0.37, SE = 0.80, z = -1.25, *p* = 0.21), but LLMs detected non-emergency cases more often (OR = 3.01, SE = 0.00, z = 358.9, *p* < 0.001) and self-care cases less often (OR = 0.25, SE = 0.00, z = -449.8, *p* < 0.001) in representative compared to traditional vignettes. The safety of advice from LLMs was higher with representative vignettes compared to traditional vignettes (OR = 3.41, SE = 0.39, z = 3.19, *p* = 0.001). Overall, LLMs were more likely to overtriage with representative vignettes (OR = 2.66, SE = 0.43, z = 2.30, *p* = 0.022). A summary can be found in Table 7.

**Table 7:** LLM’s self-triage performance with representative versus traditional vignettes, M = mean, SD = standard deviation.

Metric	Representative Vignettes, M(SD)	Traditional Vignettes, M(SD)	OR	p Value
Average Accuracy	67.6 (2.5)	57.8 (9.4)	1.52	.03
Emergency Accuracy	50.0 (35.4)	66.7 (36.5)	0.37	.21
Non-Emergency Accuracy	95.3 (3.8)	88.0 (17.9)	3.01	< .001
Self-Care Accuracy	6.15 (10.0)	18.7 (17.3)	0.25	< .001
Safety of Advice	95.6 (3.5)	87.1 (14.5)	3.41	.001
Inclination to overtriage	86.5 (11.0)	73.6 (23.5)	2.66	.022

Because all LLMs provided solutions for all vignettes, all LLMs were tested with vignettes of the same item difficulty. For representative vignettes the average item difficulty was M = 0.68 (SD = 0.42) and for traditional vignettes the average item difficulty was M = 0.58 (SD = 0.34). CCS values and ranks for each LLM are summarized in Table 8.

**Table 8:** Capability comparison scores (CCS) and ranks for LLMs in both vignette sets.

Large Language Model	Capability Comparison Score in representative vignette set	Capability Comparison Score in traditional vignette set	Rank in representative vignette set	Rank in traditional vignette set
GPT-4 (ChatGPT)	51.8%	53.3%	1	1
Llama 2	50.7%	42.2%	2	5
PaLM 2 (Google Bard)	49.6%	48.9%	3	4
Pi	49.6%	53.3%	4	2
Claude 2	48.4%	52.2%	5	3

## Discussion

In this study, we aimed to extend Brunswik’s representative design principles to self-triage audit studies and used this approach to generate a new set of vignettes for studying self-triage performance of laypeople, SAAs, and LLMs through a representative design approach and the RepVig Framework. An extensive pool of case descriptions was sampled in the target environment and provided a robust basis for randomly selecting cases, which were then stratified by symptom clusters to obtain a final set of vignettes that is representative also in terms of the distribution of cases. Then, we examined whether these vignettes result in different performance patterns than traditional vignettes. That was the case for all agents we tested, that is, for laypeople, SAAs, and LLMs, which we discuss in turn.

Although average accuracy of laypeople was similar for both vignette sets, they were more accurate in identifying emergencies and non-emergencies, and less accurate in identifying self-care cases. The safety of advice was also significantly higher (albeit with more overtriage errors, i.e., rating symptoms as more urgent than they are). From an ecological rationality perspective, these findings make sense: because individuals face no harm in calling a general practitioner but might face negative health outcomes if they experience symptoms that require treatment but do not seek health care, they can be expected to be risk averse and seek care rather than stay at home. However, this might lead to a high burden on primary care clinics. Unlike a previous study, which used traditional vignettes and reported that laypeople have difficulty identifying emergencies^47^, they identified emergencies more reliably and had difficulty identifying self-care cases when using representative vignettes. Since representative vignettes have a higher generalizability, laypeople seem more likely to identify emergencies in the real world as such cases might be clearer. Thus, from an applied perspective, decision support tools should focus on helping laypeople identify self-care cases rather than emergencies. This might help distributing resources in the healthcare system more efficiently and freeing up resources in primary care (and emergency departments).

For SAAs, we observed higher accuracy overall and for each triage level when using representative vignettes. However, we also found a slightly lower safety of advice compared to the traditional set, whereas our findings regarding inclination to overtriage are inconclusive. When comparing different SAAs using the CCS, rankings varied significantly between traditional and representative vignettes. This highlights the crucial role of the vignette sets in SAA testing. When selecting an SAA for general use in guiding patients through the healthcare system, these different vignettes would lead to varying choices.

In LLMs, we observed significant differences across all estimates when comparing representative and traditional vignette sets. The average accuracy was higher with representative vignettes, but the distribution across different triage levels varied. Emergency and self-care cases were less frequently identified correctly, while non-emergency cases were identified more often. The advice was also safer with representative vignettes, although they led to more overtriage errors. The ranking of LLMs also changed when using representative vignettes.

Overall, we found that our results differed when using representative vignettes, aligning with our hypothesis and findings from several other empirical studies^24,27,29,31^. This suggests that employing a representative design approach is valuable for evaluating laypeople’s self-triage performance with and without SAAs. Importantly, we do not claim that the sampling method presented in this article is or should be the only and best way to evaluate SAAs. On the contrary, in line with the concept of representative design, we suggest that each use case requires its specific set of stimuli (e.g., case vignettes sampled from different populations in specific situations). For example, to identify if SAAs miss severe cases with atypical clinical presentations, vignettes should only be sampled from patients that presented with atypical symptoms. In this sense, also traditional vignettes can provide valuable insights, for instance, to examine how SAAs might perform when being used by *clinicians*. Similarly, studying ED cases (as done by Fraser et al.^48^) could help determine if patients visiting the ED might have opted for primary care had they consulted an SAA beforehand. With a sample of primary care cases, the potential effect of SAAs on the redistribution of care seeking efforts could be assessed from a systems perspective. However, there may be some cases in which researchers do not want to test how SAAs perform in the real world but how they could perform in hypothetical cases, e.g., when examining if an SAA can still solve a case when another unrelated symptom is added. In such cases, artificially constructed or edited vignettes may be more helpful than representative vignettes.

Therefore, we suggest that future self-triage evaluation studies should specify to which particular decision situation they wish to generalize and sample case vignettes from that situation instead of relying on one fixed set of vignettes independent of the use case at hand. This recommendation is especially relevant considering that LLMs (and perhaps SAAs in the future) – that are regularly updated with new data^49^ and expected to significantly impact future healthcare^50,51^ – might be trained on published vignettes or could retrieve results for these vignettes through web searches. Thus, generalizability for LLMs is limited because published vignettes and their solutions can be part of the training set, and a standardized approach to vignette sampling (without a published solution) and continuously generating new vignettes may be more beneficial than using a fixed set of vignettes. Interestingly, by using more specific vignettes, researchers will ultimately provide more generalizable insights for clearly defined use cases. To aid evaluators in such endeavors, the appendix contains a sheet that guides them through the described steps to develop vignettes that are representative of an intended use case.

The approach presented in this article is particularly pertinent to achieving the general goal of SAAs, which is to enable people to assess symptoms without a clinician’s direct involvement. Beyond that use case, we propose our RepVig Framework based on the representative design approach as a standard for sampling vignettes from situations to which one wishes to generalize. For regulators evaluating SAAs and other digital health technologies, our study emphasizes the importance of data quality. Testing decision support systems should be done both with relevant metrics^36^ *and* with reliable vignettes that allow generalizations beyond the testing scenario. Because using unrepresentative vignettes for system testing can significantly influence the results – potentially leading to biased outcomes – only using representative vignettes and providing information on the vignettes that SAAs are tested with is particularly important. Stakeholders should specify the population they want to generalize to and use corresponding vignettes for evaluations.

This study has several limitations. First, we could not obtain additional information for these cases. Although this is also a common issue with traditional vignettes, having extra details available that SAAs might inquire about would be beneficial. This way, inputters get more precise results because they are able to respond to SAA questions throughout the interaction. However, interviewing individuals in the situation of experiencing the symptoms to get additional information poses significant challenges; it is particularly unfeasible and unethical for those with urgent symptoms. Therefore, this trade-off between realism and practical and ethical considerations we propose is likely the most acceptable balance achievable. A second limitation pertains to the results obtained from LLMs. These results are based on a specific prompt, and real-world interaction might yield different results due to varying prompts, interactions, and interpretation of LLM outputs. Additionally, LLM output is non-deterministic^52^, meaning results could vary even if cases are entered using the same prompt again. Given rapid improvement cycles, the accuracy of LLMs might change quickly and require ongoing tests. Next, the observed disparities in the SAAs’ inclination to overtriage are likely due to inputter variability, as it has been previously shown to affect such estimates^42^. However, we followed standard practice in SAA audit studies and this variance is present in similar studies as well. While technically all differences might stem from inputter variability, our results for laypeople and LLMs – in which inputter variability is impossible – differ between these two vignette sets too. Therefore, we believe that our results are not solely attributable to inputter variability. Another limitation of our case vignettes concerns atypical or rare cases. Because we aimed to generate a vignette set that is representative of the cases that SAAs are approached with, atypical cases or very rare cases are potentially not included in our vignette set and errors for these cases cannot be examined. Thus, future studies should specifically examine rare or atypical cases to find out how safe SAAs and LLMs are in these situations. Lastly, the exact reasons for differences in performance estimates cannot be clearly inferred from our study design. Although the symptom distribution of our cases should be similar to that in the real world as the cases were randomly sampled, there are several factors that could be responsible for performance estimate differences: the phrasing might have had an influence on participants’ decision-making, because the cases include subjective information like pain severity or expressed anxiety. The assigned gold standard self-triage level could have had an impact on observed differences as well, because the representative vignettes are phrased differently than traditional vignettes, and the representative vignettes might have been more ambiguous than traditional vignettes. However, to minimize this influence, we followed the procedure of previous studies (and current gold standard in SAA literature) by employing two licensed physicians that independently rated the symptoms and discussed cases in which they initially disagreed^10,17^. In the real world, assigning a triage level based on patients’ descriptions is not uncommon either and standard procedure in emergency departments in which designated triage nurses assess patients and their descriptions to determine the urgency they must be treated with^53^. Thus, internal validity should not be impaired. Although a connection between these factors (e.g., reported symptoms or the phrasing of symptoms) is expected according to Brunswik’s probabilistic functionalism – which proposes that cues such as symptoms and the way they are phrased are interrelated in the real world as well – future studies could explore these factors in more detail to find out which factors contribute to the observed differences.

In summary, we propose refining vignettes in self-triage studies to enhance generalizability of the observed performance patterns. To ground the vignette sampling process on a theoretical basis, we suggest using representative design principles for investigating self-triage of laypeople (see Fig. 1) and the RepVig Framework as a general framework for sampling case vignettes and evaluating triage performance in all kinds of use cases.

## Supplementary Information

Supplementary Information.

## Data Availability

The dataset that was collected and analyzed in this study can be found in the open data repository Zenodo: https://doi.org/10.5281/zenodo.12805048.
