# Supplementary material for: The RepVig framework for designing use-case specific representative vignettes and evaluating triage accuracy of laypeople and symptom assessment applications
Source: Sci Rep. 2024 Dec 23;14:30614. doi: 10.1038/s41598-024-83844-z (PMC11666565; doi:10.1038/s41598-024-83844-z)
Supplement: Supplementary file 1 — Supplementary Information. [file 41598_2024_83844_MOESM1_ESM.docx]

# **A Use-Case Specific Framework for Designing Representative Vignettes (RepVig) and Evaluating Triage Accuracy of Laypeople and Symptom-Assessment Applications**

# Marvin Kopka, Hendrik Napierala, Martin Privoznik, Desislava Sapunova, Sizhuo Zhang, Markus A. Feufel

# RepVig Framework Worksheet

# A) General considerations

Which use case do you examine?

________________________________________________________________
________________________________________________________________

Do you want to generalize to real-world situations?

[ ] No (no representative vignettes needed)

[ ] Yes (proceed with next point)

What is the reference class (use case or situation) you want to generalize to?

________________________________________________________________
________________________________________________________________

From where and how do you sample potential cases?

________________________________________________________________
________________________________________________________________

This represents:

[ ] the full reference class

[ ] a representative sample of the reference class

[ ] a non-representative sample of the reference class (stratification necessary)

Why is this sampling approach justified to allow generalizations to the reference class?

________________________________________________________________
________________________________________________________________

# B) Questions for sampling the vignette set from all cases

What are inclusion / exclusion criteria?

________________________________________________________________
________________________________________________________________

How were random samples drawn from the full vignette pool (and how was random sampling ensured)? How were they rated for inclusion/exclusion?

________________________________________________________________
________________________________________________________________

Were the vignettes stratified to reflect a representative proportion?

[ ] no

[ ] yes

The proportion was determined using/the quota were based on:

________________________________________________________________
________________________________________________________________

How many vignettes were excluded for which reasons?

________________________________________________________________
________________________________________________________________

Were the vignettes manipulated / edited in any way? If so, how (and why?)

________________________________________________________________
________________________________________________________________

**Table A1.** Included case vignettes.

| Symptom cluster | Case number | Link to case | Assigned urgency level |
| --- | --- | --- | --- |
| Musculoskeletal Pain | 1 | <https://www.reddit.com/r/AskDocs/comments/14enray/siderib_injury/> | Self-care |
|  | 2 | <https://www.reddit.com/r/AskDocs/comments/14fshtx/shin_pain_after_run_28f/> | Self-care |
|  | 3 | <https://www.reddit.com/r/AskDocs/comments/14icicl/21f_back_pain_migraines_eye_pressure/> | Non-Emergency |
|  | 4 | <https://www.reddit.com/r/AskDocs/comments/14i0dv6/16m_arm_hurt_when_curl_heavy_weight/> | Self-care |
|  | 5 | <https://www.reddit.com/r/AskDocs/comments/14kxdjj/swollen_foot_no_clot/> | Non-Emergency |
| Joint Pain | 6 | <https://www.reddit.com/r/AskDocs/comments/14gywqb/radiating_pain_in_leg_after_rolling_ankle_30m/> | Self-care |
|  | 7 | <https://www.reddit.com/r/AskDocs/comments/14avegt/i_cant_identify_this_shoulder_pain_please_help/> | Non-Emergency |
|  | 8 | <https://www.reddit.com/r/AskDocs/comments/14hh03i/fell_on_my_hands_and_thumb_hurts/> | 1-Day-Urgent |
| Headache | 9 | <https://www.reddit.com/r/AskDocs/comments/14j1dou/really_bad_anxiety_after_hitting_head/> | Emergency |
| Chest Pain | 10 | <https://www.reddit.com/r/AskDocs/comments/14epjg6/lungs_pain_inside_lung_pain/> | 1-Day-Urgent |
| Other Pain | 11 | <https://www.reddit.com/r/AskDocs/comments/14ckwcp/electric_shock_in_neck_and_armpit/> | Non-Emergency |
|  | 12 | <https://www.reddit.com/r/AskDocs/comments/14ggsg8/kidney_infection/> | 1-Day-Urgent |
|  | 13 | <https://www.reddit.com/r/AskDocs/comments/14j1jm1/ear_problems/> | 1-Day-Urgent |
|  | 14 | <https://www.reddit.com/r/AskDocs/comments/14ayz6m/why_does_my_elbow_hurt_when_i_fully_extend_it_open/> | Non-Emergency |
|  | 15 | <https://www.reddit.com/r/AskDocs/comments/14ay33z/dull_ache_in_left_testicle/> | Non-Emergency |
| Gynecological | 16 | <https://www.reddit.com/r/AskDocs/comments/14ft7ys/is_it_possible_to_have_yeast_infection_only_on/> | Non-Emergency |
|  | 17 | <https://www.reddit.com/r/AskDocs/comments/14g6ob6/period_for_25_weeks/> | 1-Day-Urgent |
|  | 18 | <https://www.reddit.com/r/AskDocs/comments/14gsz4w/no_period_in_4_months/> | Non-Emergency |
|  | 19 | <https://www.reddit.com/r/AskDocs/comments/14g48gg/bladder_and_pelvic_pain/> | Non-Emergency |
|  | 20 | <https://www.reddit.com/r/AskDocs/comments/14hjtps/f21_still_irregular_periods_a_year_later_after/> | Non-Emergency |
| Tumors/lumps/masses | 21 | <https://www.reddit.com/r/AskDocs/comments/14fn33z/weird_bump_in_my_eye_socket_should_i_be_concerned/> | Non-Emergency |
|  | 22 | <https://www.reddit.com/r/AskDocs/comments/14j1jzj/strange_mass_20m/> | Non-Emergency |
|  | 23 | <https://www.reddit.com/r/AskDocs/comments/14k8uv2/i_just_found_this_random_scar_and_lump_in_the/> | Self-care |
|  | 24 | <https://www.reddit.com/r/AskDocs/comments/14mchud/calf_knots/> | Non-Emergency |
| Edema | 25 | <https://www.reddit.com/r/AskDocs/comments/14gprss/my_leg_is_a_bit_swollen/> | 1-Day-urgent |
|  | 26 | <https://www.reddit.com/r/AskDocs/comments/14jzlhh/why_do_my_legs_do_this/> | Non-Emergency |
| Skin issues | 27 | <https://www.reddit.com/r/AskDocs/comments/14dzw38/shingles_staph_infection_other/> | Self-care |
|  | 28 | <https://www.reddit.com/r/AskDocs/comments/14ltfn7/skin_changes_on_my_thighs_photos_on_comments/> | Non-Emergency |
| Gastrointestinal | 29 | <https://www.reddit.com/r/AskDocs/comments/14l2p0e/can_someone_help_me/> | Self-care |
|  | 30 | <https://www.reddit.com/r/AskDocs/comments/14ivqxw/diahrea_for_6_weeks/> | Non-Emergency |
| Impaired sensations | 31 | <https://www.reddit.com/r/AskDocs/comments/14l11z8/left_side_of_the_lip_is_numb_like_im_at_the/> | Self-care |
|  | 32 | <https://www.reddit.com/r/AskDocs/comments/14ixbzd/19m_tingling_sensation_in_hands_and_feet_during/> | Self-care |
|  | 33 | <https://www.reddit.com/r/AskDocs/comments/14l0gge/wrist_issue/> | Self-care |
| Urinary Tract Problems | 34 | <https://www.reddit.com/r/AskDocs/comments/14jx77b/uti_or_friction/> | Non-Emergency |
|  | 35 | <https://www.reddit.com/r/AskDocs/comments/14lq0oj/weird_tingly_pressure_in_bladder_constant_urge_to/> | 1-Day Urgent |
|  | 36 | <https://www.reddit.com/r/AskDocs/comments/14e00ng/burning_pain_when_peeing_no_way_this_is_an_std/> | 1-Day Urgent |
| Upper Respiratory Symptoms | 37 | <https://www.reddit.com/r/AskDocs/comments/14i5y0o/105_fever_and_sore_abslower_belly/> | Self-care |
| Other | 38 | <https://www.reddit.com/r/AskDocs/comments/14l9bpt/blood_in_my_stool/> | Non-Emergency |
|  | 39 | <https://www.reddit.com/r/AskDocs/comments/14i3ev9/liquid_coming_out_of_my_nose/> | Non-Emergency |
|  | 40 | <https://www.reddit.com/r/AskDocs/comments/14hynsg/dog_tooth_scraped_cartridge_on_inside_of_my/> | 1-Day Urgent |
|  | 41 | <https://www.reddit.com/r/AskDocs/comments/14c6izu/should_i_go_to_er/> | 1-Day Urgent |
|  | 42 | <https://www.reddit.com/r/AskDocs/comments/14ckxmz/should_i_go_to_the_er/> | Emergency |
|  | 43 | <https://www.reddit.com/r/AskDocs/comments/14jmp9t/weird_drop_on_heart_rhythm/> | Non-Emergency |
|  | 44 | <https://www.reddit.com/r/AskDocs/comments/14jbuuu/i_inhaled_a_macaroni_noodle_and_am_scared_of/> | Self-care |
|  | 45 | <https://www.reddit.com/r/AskDocs/comments/14ly5jh/popping_lung/> | Self-care |

**Table A2.** List of included SAAs.

| Symptom Assessment Application | URL |
| --- | --- |
| Ada Health | https://apps.apple.com/de/app/ada-check-deine-gesundheit/id1099986434 |
| NHS111 online | https://111.nhs.uk/triage/check-your-symptoms |
| Drugs.com | https://[www.drugs.com/sym](http://www.drugs.com/sym)ptom-checker/ |
| Everyday Health | https://www.everydayhealth.com/symptom-checker/ |
| Family Doctor | https://familydoctor.org/your-health-resources/health-tools/symptom-checker/ |
| Healthdirect | https://[www.healthdirect.gov.](http://www.healthdirect.gov/)au/symptom-checker/ |
| Healthily | https://www.livehealthily.com/symptom-checker |
| Healthwise | https://myhealth.alberta.ca/Health/pages/symptom- checker.aspx |
| Isabel | https://symptomchecker.isab elhealthcare.com/ |
| NHS Wales | https://[www.nhsdirect.wales.](http://www.nhsdirect.wales/)nhs.uk/SelfAssessments/ |
| Symptify | https://symptify.com/ |
| Symptomate | https://symptomate.com |

**Table A3.** List of included LLMs.

| Large Language Model | URL |
| --- | --- |
| GPT-4 (OpenAI) | https://chat.openai.com |
| Claude 2 (Anthropic) | https://www.anthropic.com/claude |
| Pi (Inflection) | https://pi.ai/onboarding |
| PaLM 2 (Google) | https://ai.google/discover/palm2/ |
| LLaMa 2 (Meta) | https://llama.meta.com |

**Table A4.** SAA’s self-triage performance with representative vignettes for each inputter.

| Metric | Inputter 1 | Inputter 2 |
| --- | --- | --- |
| Average Accuracy, M (SD) | 52.0 (12.0) | 54.1 (15.0) |
| Emergency Accuracy, M (SD) | 70.8 (25.7) | 66.7 (24.6) |
| Non-Emergency Accuracy, M (SD) | 56.4 (19.5) | 66.9 (20.1) |
| Self-Care Accuracy, M (SD) | 39.1 (26.3) | 22.4 (20.3) |
| Safety of Advice, M (SD) | 83.7 (11.1) | 92.2 (5.3) |
| Inclination to overtriage, M (SD) | 52.5 (29.0) | 74.0 (17.6) |

**Table A5.** Self-triage performance of each SAA with representative vignettes.

| Symptom Assessment Application | Average Accuracy | Emergency Accuracy^*^ | Non-Emergency Accuracy | Self-Care Accuracy | Safety of Advice | Inclination to Overtriage |
| --- | --- | --- | --- | --- | --- | --- |
| Ada Health | 73.3 | 50.0 | 93.3 | 30.8 | 86.7 / 93.3 | 33.3 / 17.6 |
| NHS111 online | 80.0 | 100 | 90.0 | 53.8 | 84.1 / 93.2 | 41.2 / 33.3 |
| Drugs.com | 68.9 | 100 | 80.0 | 38.5 | 91.7 / 88.9 | 30.0 / 30.8 |
| Everyday Health | 77.8 | 50.0 | 80.0 | 76.9 | 67.5 / 95.0 | 82.4 / 13.3 |
| Family Doctor | 51.1 | 100 | 56.7 | 30.8 | 95.0 / 90.0 | 28.6 / 20.0 |
| Healthdirect | 75.6 | 100 | 83.3 | 53.8 | 78.8 / 90.9 | 60.0 / 23.1 |
| Healthily | 46.7 | 50.0 | 43.3 | 53.8 | 65.4 / 73.1 | 62.5 / 40.0 |
| Healthwise | 80.0 | 50.0 | 80.0 | 84.6 | 72.7 / 81.8 | 81.2 / 66.7 |
| Isabel | 68.9 | 100 | 96.7 | 0.0 | 100 / 97.8 | 0.0 / 6.67 |
| NHS Wales | 62.2 | 50.0 | 66.7 | 53.8 | 76.0 / 80.0 | 56.2 / 41.7 |
| Symptify | 51.1 | 100 | 66.7 | 7.7 | 96.6 / 96.6 | 6.67 / 5.0 |
| Symptomate | 77.8 | 50.0 | 80.0 | 76.9 | 67.5 / 95.0 | 87.5 / 13.3 |

^*^ Since only 2 emergency care cases were included, this point estimate is not reliable.

**Figure A1.** Performance comparison of different SAAs.

**Table A6.** Self-triage performance of each LLM with representative vignettes.

| Large Language Model | Average Accuracy | Emergency Accuracy^*^ | Non-Emergency Accuracy | Self-Care Accuracy | Safety of Advice | Inclination to Overtriage |
| --- | --- | --- | --- | --- | --- | --- |
| GPT-4 (OpenAI) | 71.1 | 100 | 100 | 0 | 100 | 0 |
| Claude 2 (Anthropic) | 64.4 | 0 | 96.7 | 0 | 93.3 | 6.7 |
| Pi (Inflection) | 66.7 | 50.0 | 93.3 | 7.7 | 95.6 | 4.4 |
| PaLM 2 (Google) | 66.7 | 50.0 | 96.7 | 0 | 97.8 | 2.2 |
| LLaMa 2 (Meta) | 68.9 | 50.0 | 90.0 | 23.1 | 91.1 | 8.9 |

^*^ Since only 2 emergency care cases were included, this point estimate is not reliable.

**Figure A2.** Performance comparison of different LLMs.
